# We can check serum lithium levels less often without compromising patient safety: evidence from a multi-centre study

**DOI:** 10.1192/bjo.2021.132

**Published:** 2021-06-18

**Authors:** Adrian Heald, David Holland, Mark Davies, Chris Duff, Ceri Parfitt, Lewis Green, Jonathan Scargill, David Taylor, Anthony Fryer

**Affiliations:** 1 University of Manchester; 2 The Benchmarking Partnership; Mike Stedman, Res Consortium; 3 Res Consortium; 4 University Hospitals of North Midlands NHS Trust; 5 St. Helens & Knowsley Teaching Hospitals NHS Trust; 6 Royal Oldham Hospital, Oldham, Pennine Care NHS Foundation Trust; 7 Institute of Psychiatry, Psychology and Neuroscience, King's College London; 8 Keele University

## Abstract

**Aims:**

Lithium was first found to have an acute antimanic effect in 1948 with further corroboration in the early 1950s. It took some time for lithium to become the standard treatment for relapse prevention in bipolar affective disorder. In this study, our aims were to examine the factors associated wtih the likelihood of maintaining lithium levels within the recommended therapeutic range and to look at the stability of lithium levels between blood tests. We examined this relation using clinical laboratory serum lithium test requesting data collected from three large UK centres, where the approach to managing patients with bipolar disorder and ordering lithium testing varied.

**Method:**

46,555 lithium rest requests in 3,371 individuals over 7 years were included from three UK centres. Using lithium results in four categories (<0.4 mmol/L; 0.40–0.79 mmol/L; 0.80–0.99 mmol/L; ≥1.0 mmol/L), we determined the proportion of instances where, on subsequent testing, lithium results remained in the same category or switched category. We then examined the association between testing interval and proportion remaining within target, and the effect of age, duration of lithium therapy and testing history.

**Result:**

For tests within the recommended range (0.40–0.99 mmol/L categories), 84.5% of subsequent tests remained within this range. Overall 3-monthly testing was associated with 90% of lithium results remaining within range compared with 85% at 6-monthly intervals. At all test intervals, lithium test result history in the previous 12-months was associated with the proportion of next test results on target (BNF/NICE criteria), with 90% remaining within range target after 6-months if all tests in the previous 12-months were on target. Age/duration of lithium therapy had no significant effect on lithium level stability. Levels within the 0.80–0.99 mmol/L category were linked to a higher probability of moving to the ≥1.0 mmol/L category (10%) than those in the 0.40–0.79 mmolL group (2%), irrespective of testing frequency. Thus prior history in relation to stability of lithium level in the previous 12 months is a predictor of future stability of lithium level.

**Conclusion:**

We propose that, for those who achieve 12-months of lithium tests within the 0.40–0.79mmol/L range, it would be reasonable to increase the interval between tests to 6 months, irrespective of age, freeing up resource to focus on those less concordant with their lithium monitoring. Where lithium level is 0.80–0.99mmol/L test interval should remain at 3 months. This could reduce lithium test numbers by 15% and costs by ~$0.4 m p.a.

